# pH-responsive polymer microcapsules for targeted delivery of biomaterials to the midgut of *Drosophila suzukii*

**DOI:** 10.1371/journal.pone.0201294

**Published:** 2018-08-09

**Authors:** Calum T. J. Ferguson, Areej A. Al-Khalaf, R. Elwyn Isaac, Olivier J. Cayre

**Affiliations:** 1 School of Biological Sciences, University of Leeds, Leeds, United Kingdom; 2 School of Chemical and Process Engineering, University of Leeds, Leeds, United Kingdom; 3 College of Science, Princess Nourah bint Abdulrahman University, Riyadh, Saudi Arabia; Biocenter, Universität Würzburg, GERMANY

## Abstract

*Drosophila suzukii* or spotted wing *Drosophila* is an economically important pest which can have a devastating impact on soft and stone fruit industries. Biological pesticides are being sought as alternatives to synthetic chemicals to control this invasive pest, but many are subject to degradation either in the environment or in the insect gut and as a result require protection. In this study we identified a sharp change in pH of the adult midgut from neutral to acidic (pH <3), which we then exploited to develop poly(2-vinylpyridine) (P2VP) microcapsules that respond to the change in midgut pH by dissolution and release of their cargo for uptake into the insect. First, we used labelled solid poly(methyl methacrylate) (PMMA) particles to show that microcapsules with a diameter less than 15 μm are readily ingested by the adult insect. To encapsulate water-soluble biological species in an aqueous continuous phase, a multiple emulsion template was used as a precursor for the synthesis of pH-responsive P2VP microcapsules with a fluorescent (FITC-dextran) cargo. The water-soluble agent was initially separated from the aqueous continuous phase by an oil barrier, which was subsequently polymerised. The P2VP microcapsules were stable at pH > 6, but underwent rapid dissolution at pH < 4.2. *In vivo* studies showed that the natural acidity of the midgut of *D*. *suzukii* also induced the breakdown of the responsive P2VP microcapsules to release FITC-dextran which was taken up into the body of the insect and accumulated in the renal tubules.

## Introduction

The invasive and polyphagous pest, *Drosophila suzukii*, is native to South East Asia, but has recently attracted much attention because of its economic impact on the soft and stone fruit industries in North America, Europe and more recently South America [1–7]. The economic damage is inflicted by the adult female laying eggs in ripening fruit by virtue of a well-developed serrated ovipositor that cuts the fruit skin for egg deposition [8]. Current control strategies rely heavily on chemical pesticides, but their use is limited by the need to avoid chemical residues on the fruit post-harvest [9–12]. Moreover, our current arsenal of insecticides is likely to shortly no longer be sufficient, due to environmental concerns and the increasing frequency of resistance. As a result it is important to consider alternatives to existing synthetic pesticides [13,14]. Biopesticides derived from natural sources with new modes of action, such as peptide/protein toxins [14–16], neuropeptides [17–20], insect hormones [21–24] and double-stranded RNA (dsRNA)[25] have been proposed as alternatives, either individually or as part of an integrated pest management strategy. Biopesticides can have a number of positive attributes such as specificity for a target pest [15], low environmental persistence [24], non-toxic residues and easy integration with other plant protection measures.

Unfortunately, many biopesticides are subject to degradation on storage, field application [16,26] and in the insect’s midgut when taken up *per os* [24,27]. Many biopesticides are also water soluble and can be easily washed from crop surfaces, resulting in reduced efficacy and potential environmental contamination [26]. For instance, spider venoms contain more than a thousand peptide toxins, many of which have insecticidal activity. The majority however are not active *per os*, due to poor uptake and rapid degradation in the digestive tract [13,28]. Where these peptides are orally active, a post-translational modification often provides increased stability, such as disulphide bonds increasing resistance to proteases [13,28,29]. Recently, the instability of ingested biopesticides and their lack of epithelial penetration have been addressed in several ways, including fusion of peptides to plant lectins [30], co-delivery with inhibitors to reduce of gut protease activity, [31,32], attachment to plant virus coat proteins [30], development of stable amphiphilic peptide mimetic analogues [24,33,34], and conjugation to polyethylene glycol (PEG) polymers [35–37].

With advances in biotechnology, numerous proteins and peptides have also been developed for therapeutic purposes, such as protein-based vaccines [38,39] and immunoregulatory peptides and proteins [40,41]. Similarly to protein biopesticides, their short half-life and poor uptake profile has limited their use. Instead of the fusion approach, where the active species is covalently linked to another peptide or protein to provide increased protection and enhanced delivery of a biopesticides, therapeutic proteins tend to be encapsulated in polymer microcapsules or embedded into nanoparticles or microgels [38,41–46]. However, in this form they do not remain active, as there is a polymeric barrier between the active species and its target and as a result, a release mechanism is required.

Commonly, polymeric carriers are made from two classes of polymers, biodegradable or acid-degradable polymers. The biodegradable polymers are based on poly(lactic acid) (PLA), poly(lactic acid–glycolic acid) (PLGA) and chitosan, and are slowly degraded with the assistance of enzymes within the body [38,41,44]. This degradation leads to a slow sustained release profile of the bioagent, over several days [41,44–46]. Acid-degradable protein-loaded vehicles tend to be either microgels or nanoparticles that are cross linked with an acid-labile acetal/ketal linkage that breaks down in mildly acidic conditions [38,39,47]. This mechanism has been successfully implemented to trigger degradation and release within the acidic phagosomes of antigen-presenting cells, disrupting the phagosomes by an osmotic pressure mechanism which releases the loaded cargo [38]. This mechanism of release is quicker, compared to biodegradable polymer based vehicles, with 80% of the loaded protein released after 5 h. However, this release rate would likely be too slow for good biopesticide efficacy, as it is longer than the midgut retention time [48].

In the present study we have developed microcapsules to deliver and protect water soluble biomaterials for delivery into the intestine of adult *D*. *suzukii*. It is known that in the related fruit fly *Drosophila melanogaster* the pH of the intestinal lumen is mostly alkali apart from a central ‘stomach’ region in the midgut. This change in pH might be exploited as an acid trigger to rapidly release water soluble bioactive compounds such as insecticidal peptides and protein toxins from pH-sensitive microcapsules for uptake across the gut wall of the insect. A similar acid region is likely also to occur in the midgut of *D*. *suzukii* which might then be exploited to efficiently deliver biological insect control agents. To this end, we have confirmed the existence of an acid ‘stomach’ in the midgut of *D*. *suzukii* and have designed pH-responsive microcapsules as a suitable delivery system for this insect pest. We have built on previous work [49,50] to use a water-in-oil-in-water emulsion as a template for the synthesis of pH-responsive polymer microcapsules. The middle oil phase in our water-in-oil-in-water emulsion precursors consist of a monomer polymerised to produce a shell of low porosity for a reasonably large molecular weight biopesticide. Using a fluorescent dextran as the cargo we demonstrate that these microcapsules can deliver and release their load in the acid region of the adult intestine for uptake into the body of the insect.

We have investigated a novel method for delivery of biomaterials to control the invasive pest *D*. *suzukii*. To do this we first investigated the changes in pH of the insect’s intestine that can be exploited to trigger release. Secondly, we investigated the maximum size of particles that the pest can ingest. Thirdly, we investigated a method to encapsulate a fluorescently tagged, water soluble model bioagent using a multiple-emulsion template. Fourthly, we investigated the triggered release from these microcapsules *in vitro*. Finally, we fed the pH-responsive microcapsules to *D*. *suzukii* adults to study their *in vivo* release properties.

## Materials and methods

### Insects

*D*. *suzukii* (an Italian strain) were maintained on a standard *Drosophila* diet (oatmeal, 7.5%; molasses, 5%; agar, 8.4%; yeast, 8.4%; methyl paraben, 0.35% in water) at 25 ^o^C in a 12:12 light-dark cycle. Females (5–10 days old) were used for the experiments apart from the particle ingestion study which used both males and females.

### Materials

Bromocresol purple, bromophenol blue, Sudan III (≥ 85% purity), methyl methacrylate (MMA), the hydrophilic emulsifier poly(vinyl alcohol) (PVA, ≥ 99% purity, mass of 65,000 Da), FITC-dextran (average mass 10,000 Da), 2-vinylpyridine (2VP, 97% purity), methyl methacrylate (MMA, ≥ 99% purity), Span 80, Brij 30, alumina and all buffer chemicals were purchased from (Sigma-Aldrich Company Ltd.) 2,2-Azobis(2-methylpropionitrile) (AIBN) was purchased from DuPont Chemicals. The monomeric MMA and 2VP were purified using a basic alumina prior to use. Milli-Q water was used at all times.

### pH profile of the intestine of *D*. *suzukii*

pH-sensitive dyes were used to determine the pH environment of the gut lumen of adult females, via incorporation of the dye into the standard *Drosophila* diet. The following dyes were used separately at the concentrations given: 0.5% (w/w) bromocresol purple (pKa 6.3) and 0.5% (w/w) bromophenol blue (pKa 3.8) [51,52]. Flies were transferred to the dye-containing diet at 7–8 days of adult life. They were allowed to feed for at least 24 h before observations were made, allowing for sufficient dye to be ingested. The flies were then chilled on ice and tissues quickly removed into water to show the stained gut, images of which were captured by optical microscopy (Leica EZ4 W). As the gut pH only remains stable for a few minutes post dissection, micrographs were taken with minimal delay [53].

### Synthesis of solid polymerised methyl methacrylate (PMMA) particles for ingestion limit determination

A polydisperse range of solid PMMA particles was produced by suspension polymerisation. In this process, we first produced an oil-in-water emulsion, where the dispersed oil phase containing the dissolved initiator was subsequently polymerised, yielding solid polymer particles. In a typical synthesis, the oil phase was comprised of: monomeric, MMA (10 ml); a thermal initiator, 2,2′-Azobis(2-methylpropionitrile) (AIBN, 126 mg); and a lipophilic dye, Sudan III (75 mg). This oil phase was emulsified using a high shear mixer (Ultra-Turrax, IKA T25) into an aqueous phase (50 ml) containing a hydrophilic emulsifier, PVA (1.5 g). The emulsion was placed in a 250 ml round bottom flask and degassed with nitrogen for 30 min before polymerisation was undertaken at 70^°^C for 4 h while maintaining stirring at 300 rpm. The resulting particles were cleaned to remove any unreacted monomer by multiple centrifugation and re-dispersion steps.

### Particle ingestion

Coloured particles were fed to flies of both sexes at an age of 7–8 days. The particles were mixed with the standard *Drosophila* diet, and the flies were kept on this diet in a plastic petri dish before observations were made. After two days of feeding, the whole intestine was dissected in water and images were taken by optical microscopy (Leica M165FC). Excreted material was analysed by first re-dispersing the faeces deposited on the surface of the petri dish in a small volume of water. The dispersed particles were then observed by optical microscopy (Olympus BX51), and the particle sizes of 700 excreted particles were analysed using Image J software.

### Synthesis of FITC-dextran loaded microcapsules from a multiple emulsion template

The W/O/W emulsion was produced via a two-step process. First, a water-in-oil emulsion was prepared as follows. An aqueous solution containing FITC-dextran (4 ml, 7 mg/ml) was injected into an oil phase composed of monomer (2VP or MMA) (10 ml), Span 80 (0.5 g), Brij 30 (1.1 g) and AIBN (128 mg). The W/O mixture was emulsified using a homogenizer (Ultra-Turrax, IKA T 25) for 5 min at 15,000 rpm at room temperature, producing a milky water-in-oil emulsion. The FITC-dextran containing W/O emulsion was then dispersed into an aqueous PVA solution (100 ml, 3 wt. % PVA) and homogenised mildly by inverting the container three times, producing a multiple emulsion. The formation of the multiple emulsion was confirmed by optical microscopy (Olympus BX51). This emulsion was subsequently polymerised in a round-bottomed flask equipped with a magnetic stirrer, reflux condenser and nitrogen inlet system. After degassing with nitrogen for 30 mins, polymerisation was carried out at 70 ^o^C for 20 h whilst agitating at a speed of 300 rpm. This yielded microcapsules, which were characterised by optical microscopy. The size distribution was determined by light scattering (Mastersizer 2000). Microcapsules were cleaned by dialysis (BioDesignDialysis Tubing, 14000 MW, Fisher Scientific) over a 5 day period to remove unreacted monomer and FITC-dextran (~ 20%) from the continuous phase.

The pH-responsive microcapsules were analysed using a Hitachi SU8230 scanning electron microscope (SEM). The microcapsules were diluted and deposited on a SEM stub and allowed to air-dry overnight. The dry microcapsules were then sputter-coated with a 4 nm layer of platinum before insertion in the SEM chamber where, they analysed at 2 kV.

### pH-dependent release of FITC-dextran from multiple emulsion templated microcapsules

The equivalent pKa of the synthesised responsive microcapsules was determined by monitoring light transmission (500 nm) through a microcapsule suspension at varying pHs. Upon dissolution of the capsules, light transmission through the suspension increases. Solutions varying in pH from 2.8 to 6 were produced from conc. HCl, and the background transmission of these solutions was measured at 500 nm. The microcapsule suspension light transmission was measured by diluting the microcapsules (50 μl) into each pH solution (2 ml), inverting the samples three times and measuring the transmission (Agilent 89090A UV/VIS). Data was fitted using OriginPro to a sigmoidal curve.

To further analyse the *in vitro* dissolution of the responsive microcapsules, continuous video recording was undertaken while citric acid was introduced to the side of the sample generating a gradual decrease in the surrounding pH. This was achieved by bringing together two separate coverslips containing the responsive microcapsule suspension and 1 M citric acid solution, respectively. Microcapsules were imaged over time as the surrounding pH decreased, and stills from this video were subsequently taken.

The *in vitro* release of FITC-dextran from the responsive microcapsules was determined by fluorescence spectroscopy in different pH environments (pH 3.8 and 6). Water-in-oil-in-water (W/O/W) templated microcapsules (2 ml) were added to solutions of differing pH (20 ml), where the low pH conditions were produced through the use of a citric acid buffer. At selected time points, samples (1 ml) were taken and passed through a 0.22 μm syringe filter to remove any undissolved capsules and solid material. The filtrate was acidified (0.1 ml of 0.1M HCl added to 0.2 ml of filtrate) to dissolve any remaining polymer, which reduces fluorescence but remains detectible. The collected samples were analysed using a 96-well black plastic plate (Corning Life Sciences) and a FLUOstar Omega (BMG LABTECH GmbH) with λ_ex_ at 485 nm and λ_em_ set at 520 nm. The collected data was normalised against standard solutions of FITC-dextran in the relevant buffers, subjected to the same processing steps as above to filter and acidify.

*In vivo* release of cargo from the pH-responsive poly(2-vinylpyridine) and non-responsive PMMA microcapsules were investigated by depositing microcapsules containing FITC-dextran to the intact surface of an organic blueberry substrate. Flies were allowed to feed on the microcapsules for 48 h before being immobilised on ice and dissected to reveal the distribution of the ingested FITC-dextran, which was captured by optical fluorescence microscopy (Leica M165FC). The presence of intact fluorescently labelled microcapsules and any dispersed non-encapsulated fluorescence in the gut lumen, internal organs and faeces was recorded.

The release and transfer of FITC-dextran into the main body of the insect was determined by extracting the haemolymph and measuring its fluorescence. Initially, flies where fed: pH-responsive, P2VP microcapsules in drosophila diet; non-responsive, PMMA microcapsules in drosophila diet; or normal *drosophila* diet, for 24 h. To extract the haemolymph flies where chilled on ice, and holes where gently made in the thorax. Three flies per replicate where then added to a 0.5 ml Eppendorf tube with 100 μl of distilled water, which was centrifuged at 2000 rpm for 1 min. The fluorescence of the resulting solution (50 μl) was analysed using a 96-well black plastic plate (Corning Life Sciences) and a FLUOstar Omega (BMG LABTECH GmbH) with λ_ex_ at 485 nm and λ_em_ set at 520 nm. The results where plotted in Prism Graphpad where a one-way Anova was performed followed by a post-hoc Dunnets test to compare means.

## Results

### Identification of an acidic region of the midgut of *D*. *suzukii* adult

**Fig 1:**
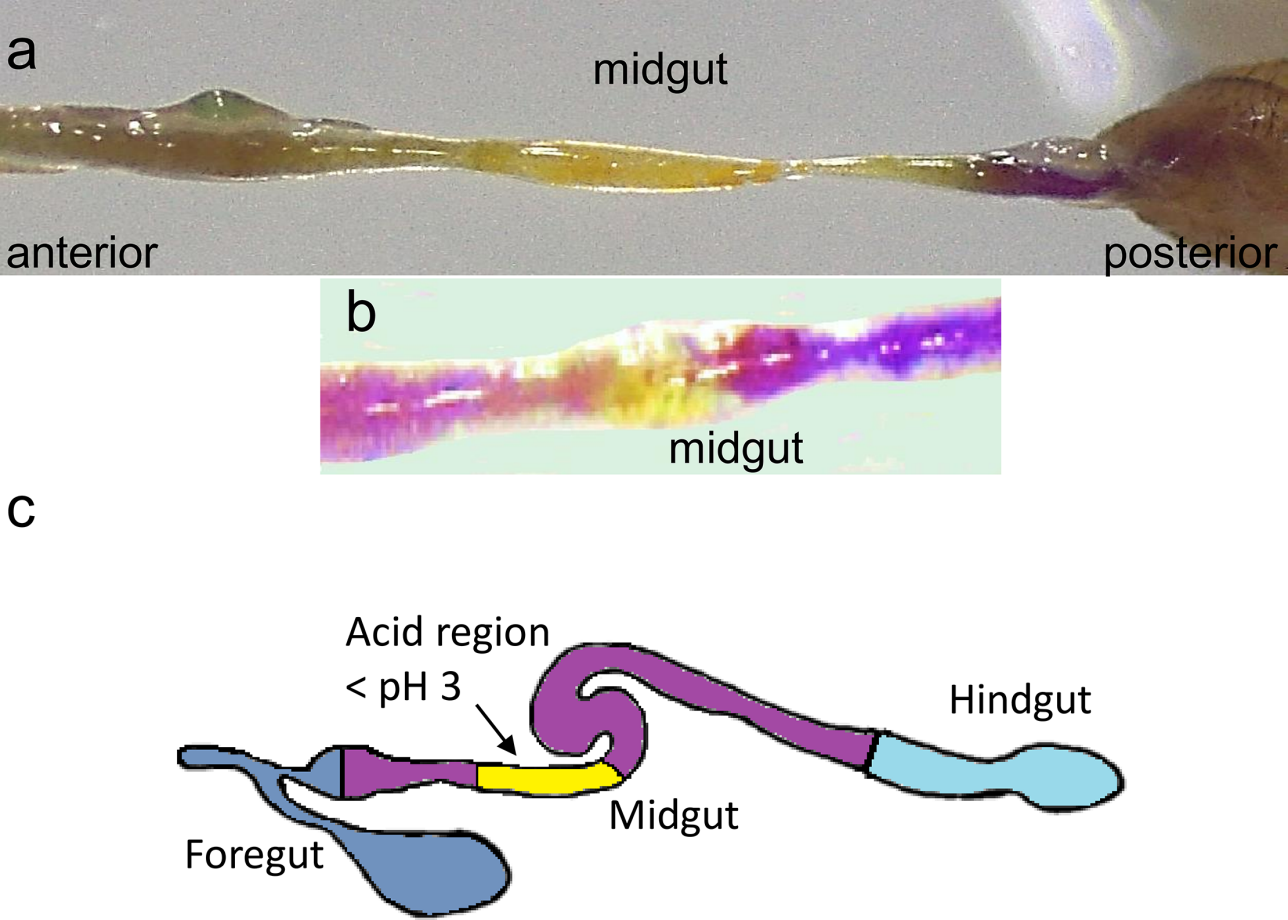
pH profile of D. suzukii midgut. The pH profile of the midgut of D. suzukii adults (7–8 days old) fed for 24 h on standard Drosophila diet containing either (**a**) bromocresol purple (0.5 w/w %) or (**b**) bromophenol blue (0.5 w/w %). In (**a**) purple indicates pH > 5.8 and yellow pH <5,2. In (**b**) yellow, pH < 3 and purple/blue, pH > 4.6. The central region of the midgut has a pH < 3, as indicated in the schematic (**c**).

After 24 h of feeding on the pH-indicator dyes, the dissected gut showed clear and reproducible staining. Bromocresol purple staining showed that much of the foregut and anterior midgut had a pH between 5.2 and 5.8, demonstrated by the dark staining (Fig 1A). A region of yellow staining (Fig 1A) was observed in the middle of the midgut showing it is acidic, pH < 5.2. The posterior midgut was stained purple with a pH > 5.8. The inclusion of bromophenol blue in the diet showed that this central region of the midgut was strongly acidic with a pH < 3 (Fig 1B).

### Particle ingestion size limit

**Fig 2:**
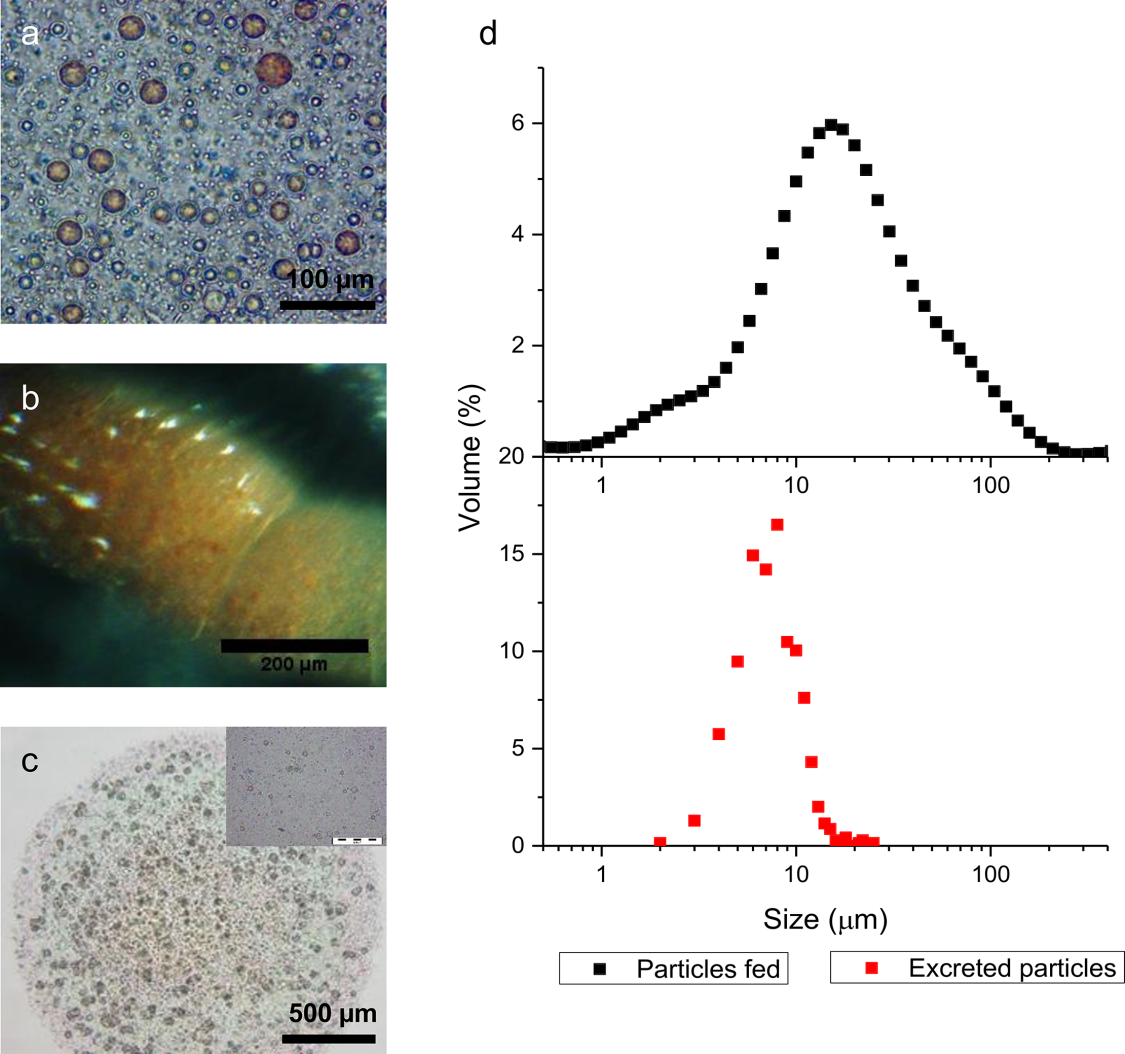
The size range of particles ingested by D. suzukii adults. (**a**) A micrograph showing polydisperse suspension of PMMA particles dyed red with Sudan III ranging in size from 0.5–200 **μ**m. (**b**) Dissected midgut of D. suzukii coloured red from ingested dyed particles but the opacity of gut hindered particle size determination. (**c**) Solid polymer particles in the fly excrement. The micrograph shows polymer particles resuspended from the excrement allowing particle size to be measured. (**d**) Comparison of the particle size range in the diet with that found in the excrement (n = 700). The maximum particle size excreted was ~ 15 **μ**m, which we have used as an approximation for the ingestion limit. Particles < 5 **μ**m were below the detection limit.

To investigate the maximum particle size that can be ingested by *D*. *suzukii* adult flies, solid PMMA particles containing the red dye (Sudan III) were produced via suspension polymerisation. Due to the random shear method used to produce these particles, the resulting samples were inherently polydisperse with a size distribution ranging from 0.5 to 200 μm (Fig 2A and 2D), measured by light scattering. These particles are clearly visible in the dissected midgut of *D*. *suzukii* 48 h after feeding on a diet containing these capsules (Fig 2B). Unfortunately, due to the opacity of the gut tissue, we were unable to accurately measure the particle size in the gut lumen, but were able to achieve this in the faeces after resuspension in water (Fig 2C). The resuspended particles were imaged using optical microscopy and the particle diameters obtained using ImageJ software.

There was a dramatic difference in the particle size distribution before ingestion and after passage through the intestine. The excreted particles have an upper size limit of around 15 μm. Particles smaller than 5 μm were poorly stained and difficult to detect in the insect’s excrement.

### W/O/W templated synthesis

**Fig 3:**
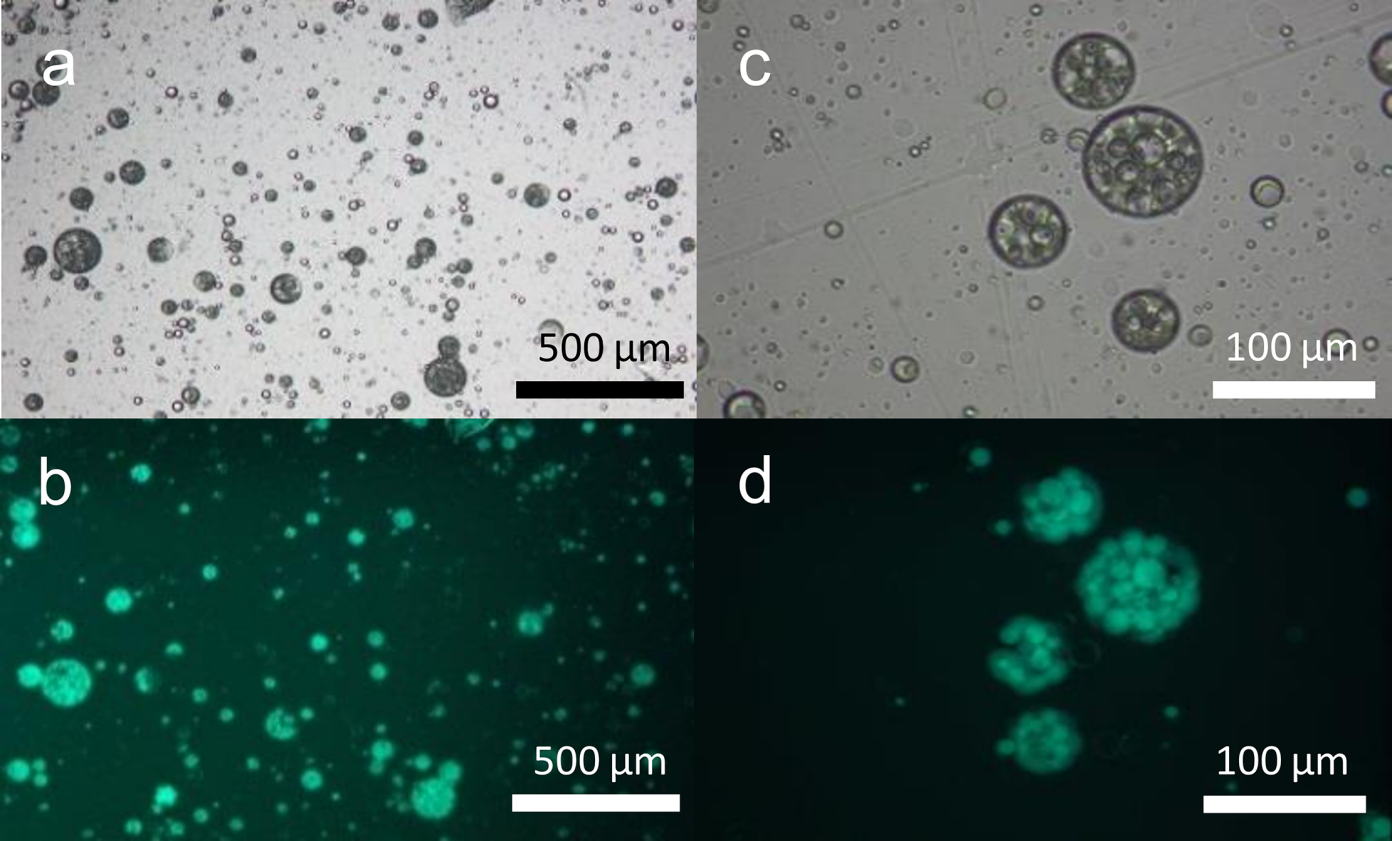
Multiple emulsion template containing FITC-dextran (10 kDa). Optical microscopy of the multiple emulsion microcapsule template prior to polymerisation (a and c) bright field images and (b and d) fluorescent images. The inner water droplets in the oil contain FITC-dextran that is localised in discrete pockets of fluorescence.

The preparation of water-core microcapsules through polymerisation of multiple emulsion templates was achieved using a methodology similar to that of Kim *et al*. [49]. Firstly, a stable multiple emulsion was obtained using a surfactant system comprising of a mixture of Brij 30 and Span 80 in the organic phase (see methods section for further details). Multiple water droplets were incorporated into the oil droplets, as observed by optical microscopy (Fig 3A–3D). The morphology of the inner water droplets was easily determined by incorporating FITC-dextran into the inner water phase. Indeed, the majority of the fluorescent material appears to have been incorporated in the multiple emulsion inner droplets (Fig 3B and 3D).

**Fig 4:**
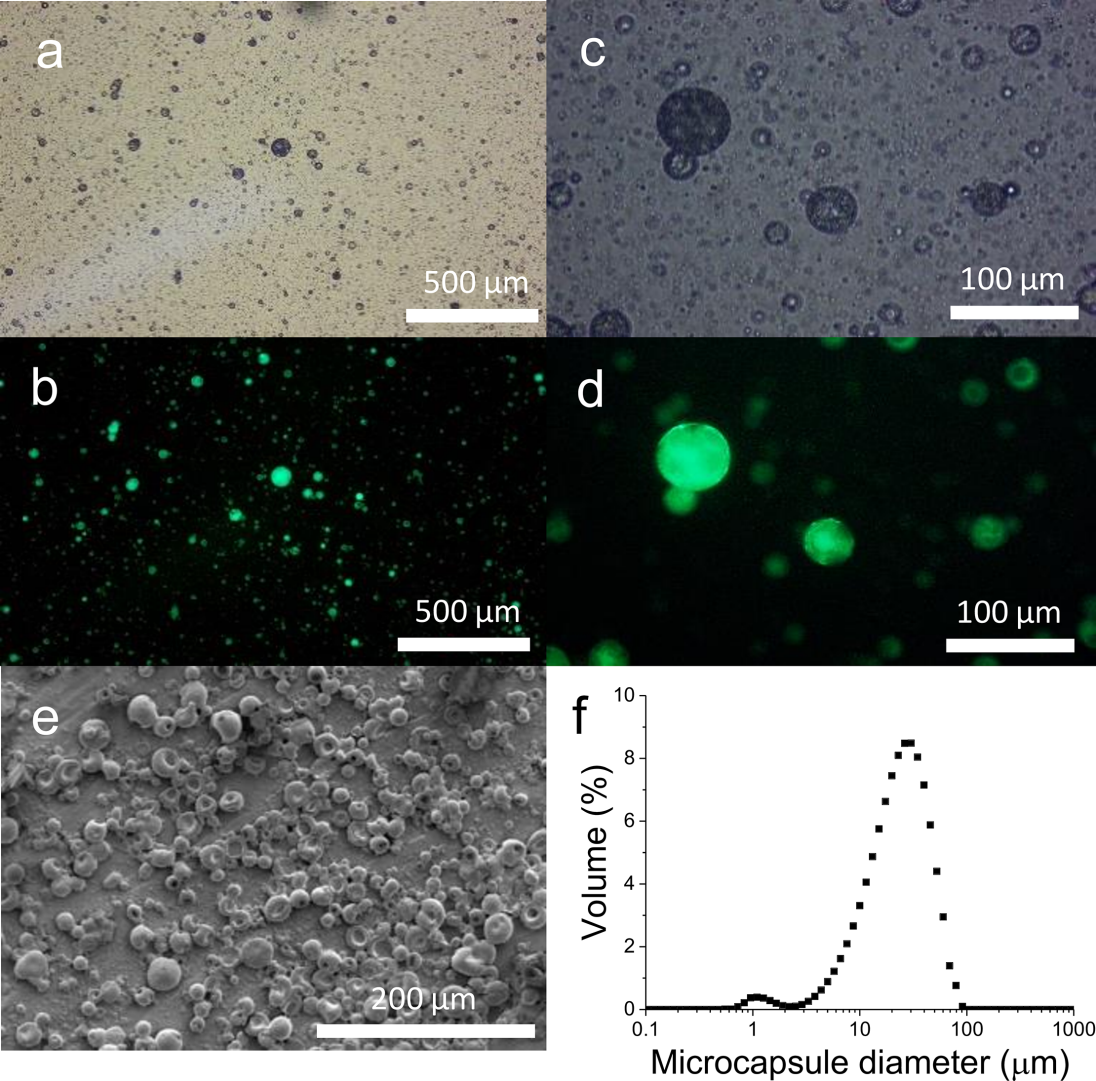
Polymerised multiple emulsion template yielding solid P2VP microcapsules. (**a-d**) Optical microscopy of microcapsules formed from polymerisation of the oil phase of the multiple emulsion template. (**e**) SEM analysis of the loaded microcapsules (**f**) Size measurement using light scattering, average distribution expressed as volume (%) (n = 10), of the microcapsules ranging from 2–100 **μ**m. The smaller peak around 1 **μ**m is a by-product formed of solid polymer particles containing no active species.

Subsequent polymerisation of the multiple emulsion template yielded solid microcapsules with encapsulated FITC-dextran (Fig 4A–4D). These microcapsules appear to have diffuse fluorescence, compared to the pockets of fluorescence observed in the emulsion, indicating that the inner water droplets had coalesced during the polymerisation process. Dialysis was used to remove non-incorporated FITC-dextran and ensure that the microcapsules used in delivering dextran to the insects was localised only within the microcapsules. SEM showed that the microcapsules had collapsed under vacuum (Fig 4E), probably as a result of loss of water during sample preparation. The method of emulsification produces polydisperse multiple emulsions, and as a result most of the microcapsules ranged in size from ~ 2–100 μm as shown in Fig 4F. A small proportion of the total particles had a diameter of less than 1 μm probably through the undesired formation of a single oil-in-water emulsion droplet during the emulsification process.

### *In vitro* release study of responsive microcapsules

**Fig 5:**
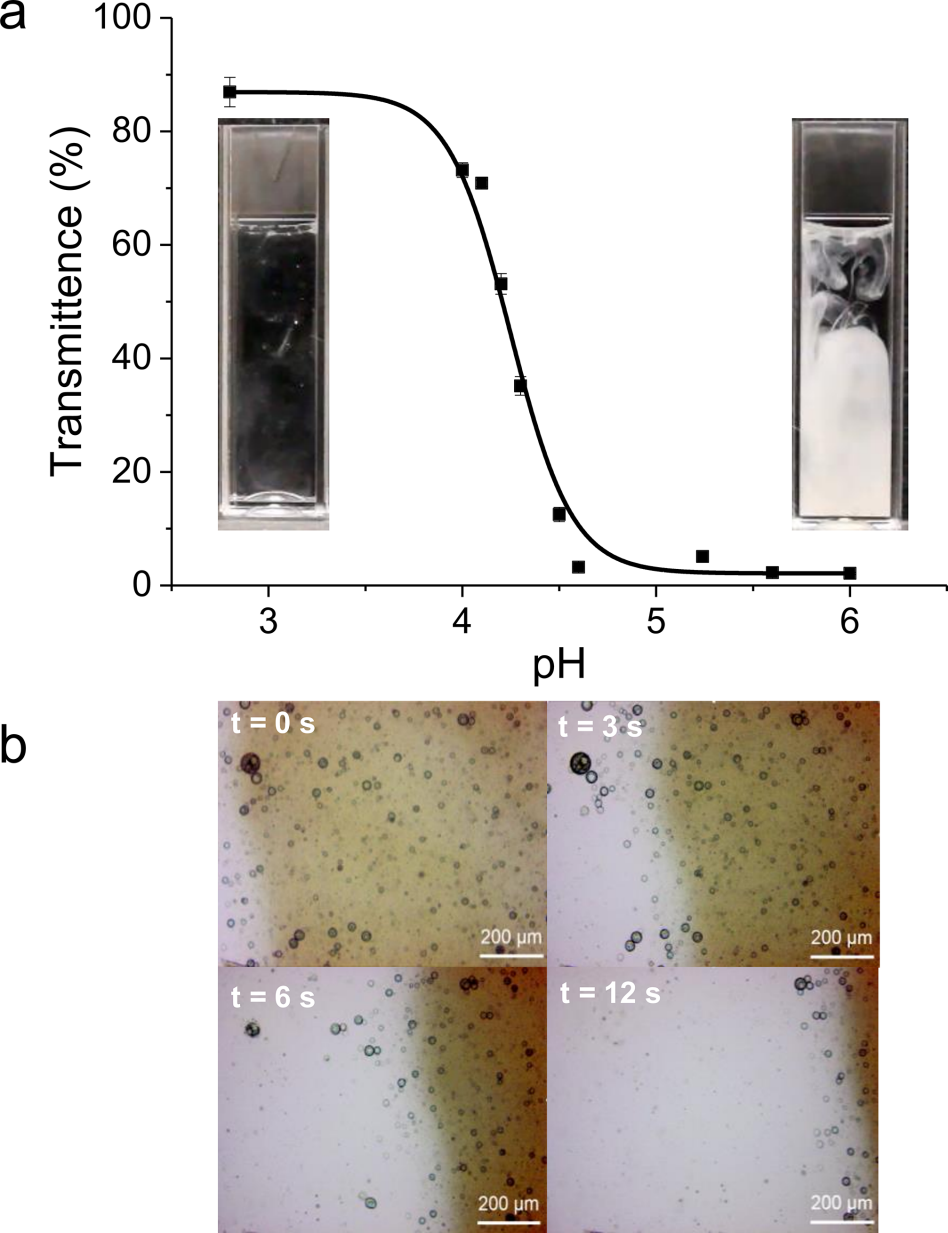
pH dependence of P2VP microcapsule dissolution. (**a**) Determination of the pKa of the microcapsules undertaken by monitoring light transmission (500 nm) as a function of pH. Data (mean±s.e.m; n = 3) were fitted to a sigmoidal curve using OriginPro 9.1 software. (**b**) Images taken at 0, 3, 6 and 12 sec from a video recording showing the effect of reducing pH on the P2VP microcapsules (see methods section) by introducing citric acid. The reduction in pH leads to swelling and rapid dissolution of the microcapsules, releasing their cargo.

The release profile of the encapsulated FITC-dextran under different pH conditions was investigated. Initially, we determined the pH required for dissolution of the microcapsules by measuring light transmission through the microcapsule suspension as a function of pH (Fig 5A). At pH > 4.5, transmission was low due to light scattering from the unchanged microcapsules. At a pH < 4, a transmission of ~ 85% was measured, which indicated that the polymer chains of the microcapsules had become protonated and disentangled, resulting in dissolution of the microcapsule shell. At intermediate pH between 4 and 4.5, a sharp change in transmission was observed. A microcapsule ‘pKa’ was estimated to be 4.2 ± 0.1 (mean±sem) by fitting a sigmoidal curve to the data in Fig 5A. The inserted images in Fig 5A show the appearance of the samples when microcapsules were added to pH 3 and 6 solutions. At pH 3 the solution was translucent with little evidence of undissolved microcapsules, in contrast at pH 6 the solution became opaque due to light scattering by the microcapsules.

To further investigate the microcapsule dissolution, light microscopy was used to observe and video-record changes in microcapsule morphology over time as the pH was gradually reduced by the introduction of citric acid at one edge of the observed sample area (Fig 5B). As the acid was introduced onto the microscope slide, the pH decreased from right to left and the microcapsules underwent an initial period of swelling followed by full dissolution. After 6 seconds, around a third of the microcapsules had disappeared, mostly the smaller ones, while the larger microcapsules were fully dissolved within 15 s.

**Fig 6:**
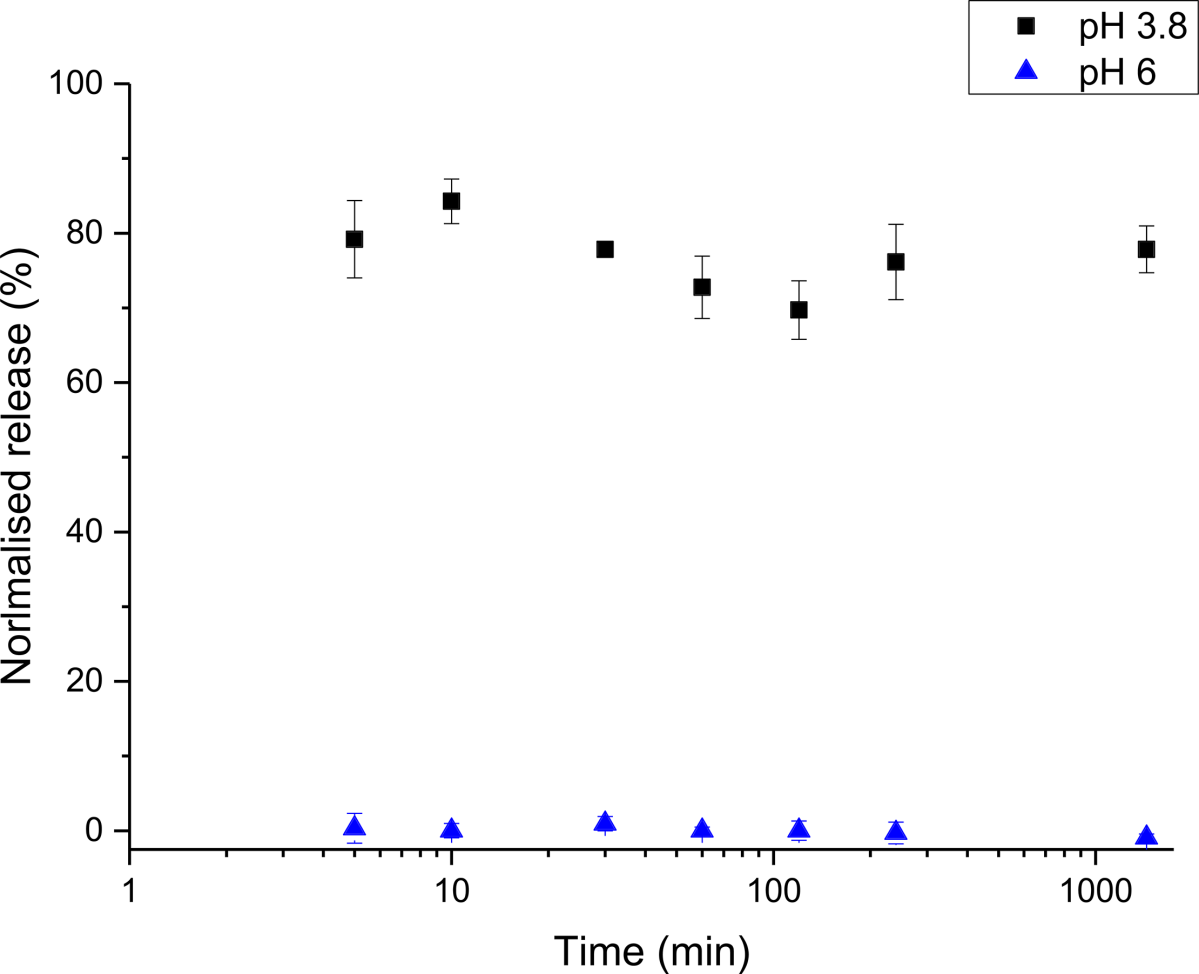
In vitro release of FITC-dextran from responsive microcapsules in different pH buffers. P2VP pH-responsive microcapsules in a pH 6 (n = 4) environment showed no release of encapsulated FITC-dextran over 24 h as the pH was above the pKa of the microcapsules. At the lower pH of 3.8 (n = 4) around 80% of the encapsulated FITC-dextran was released within the first 5 mins, no further release was observed after this time. It is assumed that the unaccounted 20% is lost during the production of the microcapsules.

We also investigated the release profile of FITC-dextran from the microcapsules as a function of time and pH, using *in vitro* tests simulating the regional pH environments of *D*. *suzukii’s* intestine. At pH 3.8 the encapsulated FITC-dextran (80% of the total dextran incorporated within the inner emulsion template water phase) is released within the first 5 mins (Fig 6). An earlier observation was not possible because of the time required to separate the continuous phase from the capsules, after 5 min there was no significant additional release. Conversely, at pH 6, which is above the pKa of the polymer, FITC-dextran was not released from the microcapsules over the same period.

### *In vivo* release

**Fig 7:**
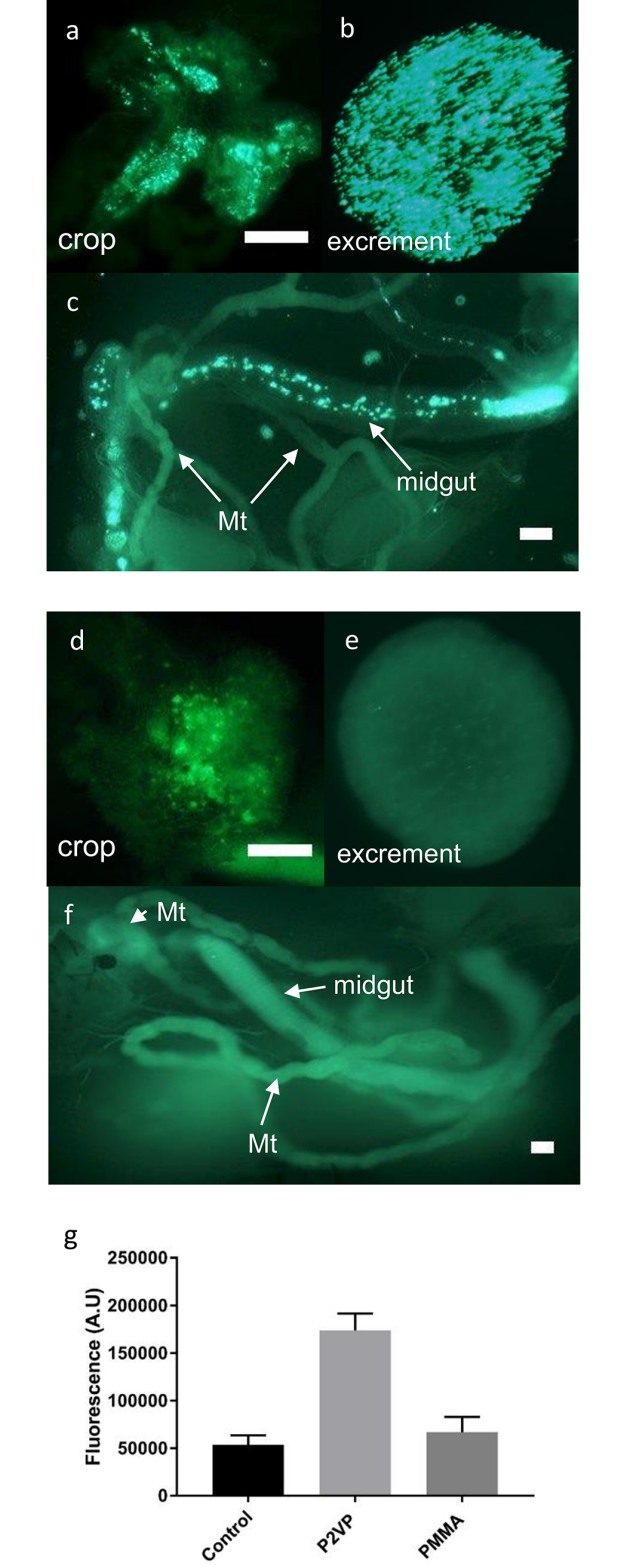
In vivo release of FITC-dextran triggered by pH changes in D. suzukii gut. In vivo release of FITC-dextran from not from control non–responsive PMMA (**a-c**), but pH-responsive P2VP (**d-f**) microcapsules fed to D. suzukii adults. Intact fluorescent PMMA microcapsules in the crop (**a**), excrement (**b**) and the lumen of the midgut (**c**). Intact pH-responsive P2VP microcapsules in the crop (**d**), but none in the excrement (**e**) and midgut (**f**). Dispersed fluorescence in the posterior midgut shows that the P2VP microcapsules have released their contents. Fluorescence was also seen in the haemolymph released from the body during dissection. Strong fluorescence accumulated in the Malpighian tubules (Mt) demonstrating uptake of FITC-dextran from the haemolymph. All scale bars 200 **μ**m. (**g**) Fluorescence in the extracted haemolymph of flies fed normal diet (control), normal diet with PMMA non-responsive microcapsules and normal diet with P2VP pH-responsive microcapsules (mean±s.e.m; n = 3). The fluorescence is significantly more intense in the haemolymph of flies fed responsive P2VP microcapsules compared to the control and non-responsive PMMA microcapsules (P = 0.0004).

Following the successful *in vitro* demonstration of the pH-dependent release of fluorescent dextran from the pH-responsive P2VP microcapsules, *in vivo* testing was undertaken to determine if the natural pH changes occurring in the gut of *D*. *suzukii* adults could trigger release of cargo from the microcapsules. Control non-responsive PMMA (Fig 7A–7C) and pH-responsive P2VP (Fig 7D–7F) microcapsules, both containing FITC-dextran, were fed to *D*. *suzukii* adult flies.

Insects fed control non-responsive PMMA fluorescent microcapsules had intact capsules in the crop, the midgut and the faeces (Fig 7A–7C). In contrast, intact microcapsules were only found in the crop of flies fed the pH-responsive P2VP microcapsules (Fig 7D). Fluorescence was seen in the midgut and in the excrement of these flies, but this was diffuse and clearly not contained in microcapsules (Fig 7E and 7F). Fluorescence was also present in the haemolymph that was released into the bathing saline used during dissection of the tissues (Fig 7F), showing that the FITC-dextran had been taken across the gut wall into the haemocoel of the P2VP microcapsule fed flies. Collected haemolymph contained significantly higher levels of fluorescence in flies fed P2VP responsive microcapsules compared to control PMMA microcapsules and flies fed on drosophila diet-only (Fig 7G). The excretory Malpighian tubules from flies fed the control microcapsules (Fig 7C) appeared dull with limited background fluorescence, whereas *per os* delivery of P2VP microcapsules led to bright fluorescence accumulating in the tubules (Fig 7F) indicating the removal and concentration of FITC-dextran from the haemolymph.

## Discussion

The gut of *D*. *suzukii* adults is divided into three distinct regions: the foregut, the midgut and the hindgut. The changes in gut pH for *D*. *suzukii* are very similar to those reported for *D*. *melanogaster*, a phylogenetically close relation and model species [51,53]. This sharp reduction in pH was exploited for a triggered release of cargo from the pH-responsive microcapsules at the main site of nutrient uptake by the insect [54–56]. To form these triggerable microcapsules, the pH-responsive monomer 2VP was chosen to be polymerised into the microcapsule shell as the pKa of non-crosslinked linear P2VP chains is in the region of 3.8–4.7, depending on chain length [57]. In acidic conditions (pH < 3.8), we expected that the protonation of the tertiary amines on the polymer chains would lead to dissolution of the entangled linear polymer chains and dissociation of the microcapsule shells, thus enabling full release of the contents [58].

Microcapsules used for the oral delivery of biopesticides should be below the size limit for particle ingestion, which can differ greatly between species. For example, the Mediterranean fruit fly *Ceratitis capitata* filters food through modified mouthparts which results in only particles of less than 0.5 μm diameter being readily ingested and passed into the intestine [59]. In contrast the decorous black fly *Simulium decorum*, which is of a similar body size, can ingest much larger particles up to a maximum of 400 μm [60]. Therefore, it was initially necessary to determine the size limit for ingested particles for the *D*. *suzukii* adults to ensure that the microcapsules were designed for the appropriate size range. The method we used to produce pH-responsive microcapsules utilised uncontrolled shear to produce both the initial and secondary emulsions and as a result the microcapsules produced were polydisperse in the size range 2–100 μm with a secondary peak evident at 0.5–2 μm, which is likely the result of nucleation and limited growth of solid P2VP particles. Indeed, during the secondary emulsion step, inevitably a small quantity of oil-in-water emulsion droplets (not containing any inner water droplets) form along with the targeted multiple emulsion droplets. The quantity of this by-product is small and as a result did not interfere with our investigations. Only a portion of the microcapsules we produced were smaller than 15 μm, and therefore further product development is desirable using more controlled emulsification techniques, such as membrane emulsification, which would decrease the multiple emulsion template size and polydispersity [61].

In order to demonstrate the potential of our microcapsules to carry and deliver water-soluble bioagents to the insect gut, we used FITC-dextran (10 kDa) as a model biological encapsulate of a similar size to some known insecticidal proteins [62]. Multiple emulsion templated synthesis leads to encapsulation of the water-soluble dextran in a water continuous phase. Polymerisation of the multiple emulsion template yielded solid pH-responsive microcapsules, where the inner oil phase was polymerised to form linear entangled P2VP chains. During this polymerisation process a change in the morphology of the inner cargo loaded water droplets occurred. Prior to polymerisation the inner water droplets appeared to have a random size distribution consistent with the sizes of the water in oil emulsion precursor. Upon polymerisation of the water phase we obtained coalescence of the inner water droplets as seen by the SEM, where the microcapsules appeared to collapse in half. It is interesting to note that upon heating of the multiple emulsion template without the presence of an initiator we did not see coalescence of the inner emulsion droplets and therefore the coalescence must result from the polymerisation of the oil phase (data not shown).

In order to investigate the physical response of the microcapsules to changes in pH, a series of *in vitro* and *in vivo* experiments were conducted. The microcapsules were shown to dissolve in a suspension upon a reduction in pH with an estimated pKa of 4.2, which is consistent with the literature values for the pKa of P2VP [57,58]. To further investigate microcapsule dissolution at low pH, we observed the microcapsules using an optical microscope as they were introduced to a low pH environment. Swelling and subsequent dissolution of the microcapsules was observed as the pH of the continuous phase around the microcapsules decreased to < 4.2. Dissolution of the microcapsules is a favourable mechanism for the release of large molecules, compared to diffusion through a swollen shell due to lower porosity or adsorption of the active onto or within the polymer shell. As a result it is important to consider the composition of the oil phase so that the resulting polymer is not crosslinked and upon reduction in pH the polymer chains are fully solubilised. *In vitro* experiments also showed at pH 3.8, that full dissolution of the microcapsules occurred with ~ 80% of the FITC-dextran released in the first 5 min, based on theoretical calculations assuming an encapsulation efficiency of 100%. No additional release of FITC-dextran was observed at low pH over the following 24 h. It is likely that the remaining ~ 20% of the FITC-dextran was lost during the formation of the microcapsules, in particular during the formation of the multiple emulsion template and the polymerisation step where transfer of the FITC-dextran from the inner water phase to the continuous phase was possible. Indeed, there is evidence for this in the formation of small solid polymer objects due the polymerisation of an oil-in-waster emulsion. Conversely, at pH 6, no release of FITC-dextran was observed over the course of the experiment, establishing that the dextran molecules were retained within the microcapsules at a pH above their pKa. Optical microscopic observations made in parallel showed no visible changes in the microcapsules at pH 6.

Further investigation was undertaken to determine if the *in vitro* release could be replicated *in vivo*, elicited by the intrinsic changes in the gut pH of *D*. *suzukii*. A *per os* study investigation was undertaken that compared the release of FITC-dextran from responsive P2VP microcapsules and non-responsive PMMA control microcapsules in the insect gut. The inclusion of non-responsive control microcapsules in our study showed that the release of fluorescence was not triggered by factors other than the gut pH, such as rupture by the insect’s mouthparts. In both cases, discrete microcapsules were observed in the crop of *D*. *suzukii* flies, showing that small microcapsules were not broken by the mouthparts. Note that the food stored in the crop had not yet reached the acidic midgut, thus both the responsive and non-responsive microcapsules remained intact. The dissection of the intestine was undertaken carefully in order to avoid damaging the tissue so that any fluorescence seen in the haemolymph and renal tubules must have been actively taken up from the gut lumen. The presence of the FITC-dextran in the haemolymph of flies fed the responsive P2VP microcapsules but not the control PMMA microcapsules was confirmed by extracting it and measuring the fluorescence. Control non-responsive PMMA microcapsules were easily identified in the lumen of the acidic midgut, demonstrating that they remain unaltered by the reduction in pH. In contrast, a band of soluble non-encapsulated fluorescence was observed in the midgut of the insect when fed the responsive P2VP microcapsules, indicating that the microcapsule contents had been released. Moreover, the renal (Malpighian) tubules were brightly fluorescent, showing that the FITC-dextran had crossed the gut wall and transferred to the haemolymph before being excreted via the tubules. By extracting the haemolymph of flies we were able to confirm the release and uptake of the FITC-dextran from pH-responsive microcapsules, but not in non-responsive microcapsules.

All these observations confirmed that the responsive microcapsules successfully dissolved in the midgut and released the FITC-dextran, which subsequently was taken up into the fly’s body in a manner typical to other foods. Finally, comparison of the excrement showed striking differences between flies fed the control and responsive microcapsules. Control non-responsive PMMA microcapsules were seen clearly in the excrement and appear to be unaltered by the digestive juices of *D*. *suzukii*. In contrast there were no intact pH-responsive P2VP microcapsules in the excrement; instead fluorescence was seen evenly distributed throughout the faeces, confirming that they had been degraded during passage through the intestine releasing their soluble FITC-dextran cargo.

In this study we have developed and tested a pH-responsive microcapsule suitable for the protection and delivery of water-soluble biopesticides. We have exploited the sharp change in pH from neutral to acid that occurs in the midgut of *D*. *suzukii* adults to trigger the release of a cargo in a region of the intestine critical for nutrient adsorption [54–56]. These microcapsules offer a smart technology to deliver water soluble control agents such as peptide hormones, protein toxins and nucleic acids that are normally unstable in the field and the gut of the spotted wing drosophila, *D*. *suzukii*.
